# Stress Exposure in Significant Relationships Is Associated with Lymph Node Status in Breast Cancer

**DOI:** 10.1371/journal.pone.0149443

**Published:** 2016-02-24

**Authors:** Chiara Renzi, Valeria Vadilonga, Sara Gandini, Giada Perinel, Nicole Rotmensz, Florence Didier, Maria Rescigno, Gabriella Pravettoni

**Affiliations:** 1 Applied Research Division for Cognitive and Psychological Science, European Institute of Oncology, Milan, Italy; 2 Division of Epidemiology and Biostatistics, European Institute of Oncology, Milan, Italy; 3 Immunobiology of Dendritic Cells and Immunotherapy Unit, European Institute of Oncology, Milan, Italy; 4 Department of Oncology and Hemato-oncology, University of Milan, Milan, Italy; Shanghai Jiao Tong University School of Medicine, CHINA

## Abstract

**Objective:**

Life stress exposure may impact on health and disease. Previous literature showed that stressful life events are associated with cancer incidence, survival and mortality. In animal models, patterns of maternal care have been shown to critically affect stress sensitivity and immunity trajectories later in life, by modifying DNA methylation during critical periods early in life. However, the role of parental care in breast cancer progression and survival has only limitedly been explored. Here, we investigated whether these factors may be linked to biological prognostic variables.

**Methods:**

One hundred twenty-three women hospitalized for surgery of primary breast cancer completed a questionnaire assessing parental bonding. Stressful events throughout the life span were also assessed.

**Results:**

We found that the absence of optimal parental relationships is significantly associated with an increased risk of lymph node involvement, adjusting for confounders, while cumulative stress in the area of sentimental relationships is borderline significantly associated with the same prognostic factor.

**Conclusions:**

Our results suggest that parental bonding and sentimental relations may have a role in breast cancer progression. These variables represent an important evolutionary aspect which may modulate cancer progression through psycho-physiological stress pathways and influence the immune system.

## Background

Exposure to stress throughout life may increase vulnerability to disease development [1,2]. In fact, when the perceived intensity of an event or a situation goes beyond the individual’s resources (such as coping style and social support), the nervous system activates information-processing pathways which result in the release of cathecolamines, corticosteroids, opioids, and inflammatory molecules. These, in turn, can influence the efficacy of the immune system e.g., by modulating the activity or the number of natural killer cells or cytotoxic T lymphocytes (see [3] for a review). Many of these physiological pathways are also relevant to the growth and metastasis of cancer. For instance, cathecolamines and glucocorticoids seem to have a role in determining the tumor microenvironment [3]. Therefore, it has been hypothesized that life stress exposure may play a role in the onset and progression of cancer (see e.g.,[4–6]). Conflicting evidence has been reported on the relation between stress exposure and different types of cancer. Overall, the results of three meta-analyses supported a modest association between psychosocial factors and breast cancer incidence, disease-free survival, and mortality [7–9]. For instance, Chida and coworkers [7] found that stress derived from life events was negatively associated with cancer survival and positively associated with mortality. Furthermore, poorer prognosis of breast cancer was significantly linked to overall stress-related psychosocial factors (13% increase in hazard ratio).

Parental care may also have a role in determining vulnerability to disease, including cancer development and progression [10]. In fact, early stress experienced through attachment relations may sensitize the individual to potential stressors later in life [11], and determine how life events have an impact on cancer-related pathways.

In animal models, patterns of maternal care have been shown to critically affect the functioning of the HPA axis and immunity trajectories later in life [11–14]. For instance, the absence of the mother during critical time windows leads to lower levels of CD8 cells and NK cells activity in young monkeys compared to pups grown under normal conditions, a pattern which could not be reversed later in life [14]. Parental care may exhibit such long-term effects by modifying DNA methylation during early critical periods [11–13]. In humans, attachment relations exert a pivotal role in determining the child’s response to stressful or frightening experiences, and critically affect emotional regulation in adulthood [15]. Cross-sectional and longitudinal studies reported that individuals who described their parents as less close, loving and protective were at higher risk of developing cancer [16–18]. In accordance, women with breast cancer obtained significantly higher scores on avoidant attachment and emotional control compared to healthy women [19].

Non-optimal parental relationships were associated with cancer incidence, but their role in cancer progression has only limitedly been investigated. Life events have been previously associated with breast cancer survival and mortality, however it is not clear whether such association is mediated by parental relations and/or by biological pathways. To our knowledge, no study assessed stress exposure by taking into consideration both parental relations and life events throughout the whole life span, in relation to prognostic factors (e.g., stage, vascular invasion, lymph node involvement) for cancer. Here, we explored whether parental bonding and life events were associated with prognostic variables in a sample of women hospitalized for surgery of primary breast cancer. In line with a consistent body of research indicating that stress has an influence on physiological mechanisms involved in metastatic processes [3], we hypothesized that stress derived from early dysfunctional parental relations and repeated stressful life events may have a role in the prognosis of breast cancer.

## Methods

Patients with histologically confirmed diagnosis of breast cancer were identified via two databases: the Institutional breast cancer Database and the Tumour Registry of the European Institute of Oncology. Written informed consent was obtained from patients taking part in the study. The study was approved by the European Institute of Oncology Institutional Review Board.

### Selection of patients

Inpatients were hospitalized in the Senology Unit of the European Institute on Oncology in Milan, Italy. All women were diagnosed with primary breast cancer and underwent quadrantectomy or mastectomy as a first therapeutic approach between July 2011 and December 2012. Exclusion criteria were major psychiatric diseases or severe neurological events that would have interfered with language comprehension or completion of the measures, prior history of cancer, and diagnosis of benign disease at the histopathological exam. Table 1 shows the main demographic and medical characteristics of the sample.

**Table 1:** Socio-demographic and medical features of all patients included.

Variables	Categories	Frequency	Percent
Age	≥50 y	64	52%
	<50	59	48%
BMI	≥25	42	34%
	<25	81	66%
Education	Elementary and middle school	29	24%
	High school	55	44%
	University	37	30%
	Missing	2	2%
Marital status	Married/living with partner	94	77%
	Divorced/single/widow	26	21%
	Missing	3	2%
Working status	House-wife, unemployed, retired	24	20%
	Actively working	96	78%
	Missing	3	2%
Parity	No	25	20%
	Yes	98	80%
Breast cancer family history	No	73	59%
	Yes	50	41%
Invasive status	*InSitu*	8	6%
	Invasive cancer	115	94%
Surgical type	Mastectomy	13	11%
	Quadrantectomy	110	89%
Tumor Type*	Her2Pos	6	5%
	LuminalA	34	29%
	LuminalBHer2Neg	53	46%
	LuminalBHer2Pos	8	7%
	TripleNeg	14	12%
Lymph-node status*	Negative (N0)	58	54%
	Positive (N1)	57	46%
pT*	1a	2	2%
	1b	9	8%
	1c	57	49%
	2	44	38%
	3	3	3%
G*	Missing	2	1%
	I	11	10%
	II	46	40%
	III	56	49%
Vascular invasion*	Absent	77	6%
	Present	37	32%
	Missing	1	2%

* excluding *InSitu*

One hundred sixty-seven patients were approached in the Unit by a clinical psychologist or by a research assistant and asked to participate in the study. A total of 162 women agreed to participate (five women refused due to lack of time, fatigue or pain related to post-operative complications). In this case, written informed consent was obtained after explaining the content of the evaluation. Of those, thirty-nine patients did not complete all measures or had partially completed questionnaires and where thus considered as drop-outs. A total of 123 women were available for complete data analysis. Patients were enrolled during hospitalization, in the days following surgical treatment. In the majority of cases, tests were completed during the permanence in the hospital. When this was not possible, an appointment was scheduled on the same day of surgical follow-up (within a week from discharge). Patients had not received histopathological results at the time of assessment.

### Measures

Demographic data and life-style variables were recorded in a case record form.

#### Parental relations

The Italian version [20] of the *Parental Bonding Instrument* (PBI; [21]) is a self-administered questionnaire measuring perceptions about parental behaviors received from childhood to adolescence (until 16 years of age). This is a 25 item scale, which in its original form identifies two dimensions: one relative to parental *care* (12 items) and the other relative to *overprotection* or *control* (13 items). The individual has to evaluate the degree of accordance of the sentences presented with respect to her/his subjective experience with maternal and paternal figures. Responses are rated on a 4-point Likert-scale (0 = Very unlike; 3 = Very like). Based on the combination of scores obtained in the care and control dimensions, each parent can be assigned to one of four styles: “*affectionate constraint*” is characterized by high care and high control/protection; “*affectionless control*” by high control/protection and low care; “*optimal parenting*” by high care and low protection/control; “*neglectful parenting*” by low care and low protection/control. Assignment to ‘high’ or ‘low’ category is based on cut-off scores differentiated for maternal and paternal figures.

In accordance with the principles of attachment theory [15], parental caregiving is supposed to have a critical role in determining the attachment relationship. Therefore this measure is considered as an indicator representing the quality of attachment [21]. For instance, a parent which is perceived as emotionally close, loving and not controlling will favor the development of a secure attachment. The PBI showed convergent validity with the Adult Attachment Interview, for what concerns optimal relations/secure attachment [22].

#### Life stress events

The Italian version [23] of the *Interview for Recent Life Events* scale [24] was used to assess the frequency and impact of stressful life events. Differently from the original instructions, the investigation included events throughout the life-span. The interview was completed together with a clinical psychologist or a trained research assistant. The scale consists of 61 life events which can be further divided into ten areas (work, education, financial problems, health, bereavement, emigration, sentimental life, legal issues, family relations and marital relations). We included an additional separate area with those items concerning stress related to pregnancy status. Patients were asked to indicate how many times in their life they experienced each event, the year when the event occurred and to rank its impact.

#### Statistical analysis

Descriptive statistics (median and interquartile ranges—IQR) and frequencies were used to describe patients’ socio-demographic features and relevant clinical variables.

Differences in frequencies of patients by life stress events and parental styles were evaluated in association with breast cancer prognostic factors using Chi-square tests for independence of categorical variables. Differences for continuous variables were assessed by Wilcoxon tests.

The variable “Parental relations style” was categorized considering optimal relations versus other types. Frequencies of patients for each stress category (work, sentimental, etc.) were evaluated considering more than one occurrence of at least an event in the category versus one or zero occurrences.

Associations with each prognostic factor coded in categorical variables (invasive status, lymph node status, tumor burden, vascular invasion, histological type, hormonal receptor status, Ki-67) were investigated through logistic regression models, adjusting for possible confounding factors (age, BMI, menopausal status, family history, parity, education, marital status) and other prognostic factors. When prognostic factors were indicated by variables that can be considered as continuous measures (e.g., Ki-67 or tumor burden), Wilcoxon signed-rank test and ANCOVA models were evaluated. Residuals from full model were checked to verify normal distribution.

First of all, the analysis was carried out considering invasive status (*InSitu* vs. invasive cancer) as response variable. Then, all the associations with the other prognostic factors were evaluated excluding cases classified as *InSitu*.

Results from the final model including significant factors are shown (see also data in S1 Dataset). Odds ratios (ORs) assessing significant associations of stress events categories and parental styles with prognostic factors are presented with 95% Confidence Intervals (CI). Two-sided *P*-values were used in the analyses. The criterion for statistical significance was set at 5%. Data were analyzed using the SAS System Software for Windows, release 8.0. (SAS Institute, Cary, NC, USA).

## Results

Socio-demographic and medical characteristics of the sample are presented in Table 1. Women had a mean age of 50 years (ranging from 28 to 65 years of age). Seventy-five percent had at least a high school diploma. Seventy-seven percent of the sample was married or had a stable relationship, while 20% was single (never married, separated, divorced or widowed). More than 40% had a family history of breast cancer. Invasive cancers were the majority (94%; *InSitu*: n = 8).

Median age was not different by lymph node status: 50 (IQR: 45–56) and 51 (IQR:45–57) for negative and positive lymph-node status respectively (*p* = 0.10).

**Fig 1:**
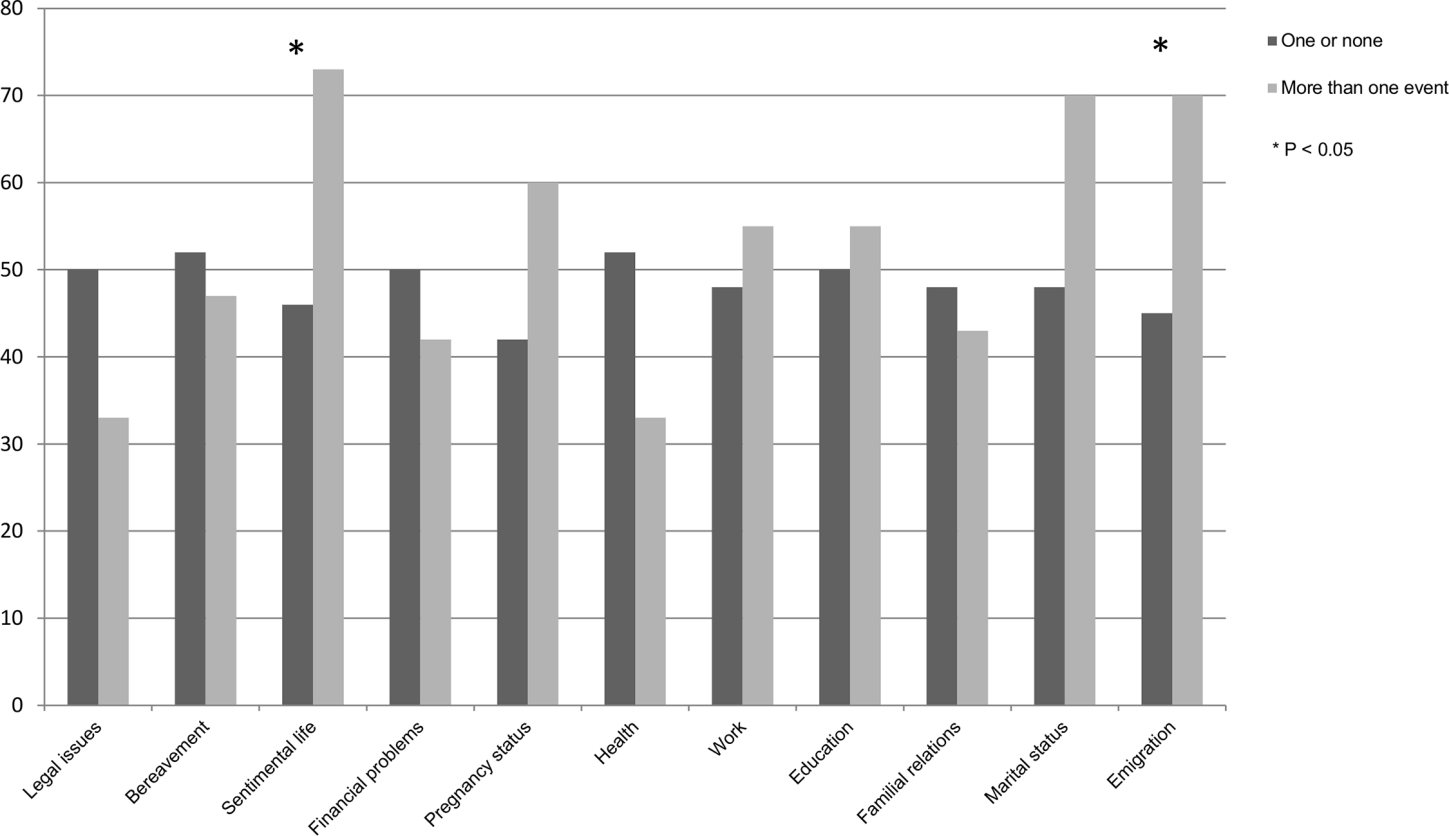
Histogram of frequencies (%) of patients with positive lymph node (N1) status by category of life stress events. Dark bars depict percentage frequencies of N1 patients who experienced repeated stress (at least one event of the category occurred more than once), light bars depict percentage frequencies of N1 patients who did not experience repeated stress (all events in the category occurred once or never). Asterisks indicate significant P-values derived from Chi-squared test.

Table 2 and Fig 1 present frequencies of patients with invasive cancer by type of stress life events (zero or one event vs. more than one event) and lymph-node status (positive or negative) with P-values from Chi-square test, showing that patients who experienced repeated stress in the area of relations or in the area of emigration were more likely to have a positive lymph node status.

**Table 2:** Frequencies of patients by type of stress life events and lymph-node status. Significant P-values are indicated in bold.

				N0		N1		
		Tot		n.	%	n.	%	
**All**		115		58	50	57	40	
Legal issues	0–1 event	112	100%	56	50	56	50	0.57
	>1 events	3	100%	2	67	1	33	
Bereavement	0–1 event	54	100%	26	48	28	52	0.64
	>1 events	61	100%	32	53	29	47	
Sentimental life	0–1 event	100	100%	54	54	46	46	**0.05**
	>1 events	15	100%	4	27	11	73	
Financial problems	0–1 event	103	100%	51	50	52	50	0.56
	>1 events	12	100%	7	58	5	42	
Pregnancy status	0–1 event	92	100%	56	58	42	42	0.09
	>1 events	23	100%	10	40	15	60	
Health	0–1 event	103	100%	50	49	53	52	0.23
	>1 events	12	100%	8	67	4	33	
Work	0–1 event	93	100%	48	52	45	48	0.60
	>1 events	22	100%	10	45	12	55	
Education	0–1 event	102	100%	52	50	50	50	0.77
	>1 events	13	100%	6	46	7	54	
Familial relations	0–1 event	76	100%	38	52	38	48	0.60
	>1 events	39	100%	20	57	19	43	
Marital status	0–1 event	105	100%	55	52	50	48	0.18
	>1 events	10	100%	3	30	7	70	
Emigration	0–1 event	95	100%	52	55	43	45	**0.04**
	>1 events	20	100%	6	30	14	70	

When models adjusted for confounders were considered, we could confirm that the proportion of patients with positive lymph node status is borderline significantly greater in patients with a history of sentimental stress events (at least one event in the category occurred more than once) than in patients with no repeated stress events in this area. Stress life events related to emigration were not any more significantly associated with lymph node status. Other life stress events categories were not significantly different by lymph node status. No association was found with other relevant prognostic factors.

Considering all the categories of parental style, no significant association was found with any of the prognostic factors. When the”parental relations style” (see S1 Table) was categorized in optimal (N = 51, 44%) versus other (N = 64, 56%) for the relation with the mother or the father (at least one optimal parental relation vs. no optimal parental relation), we found a statistically significant association with lymph node status.

Odd Ratios and 95% CI from multiple logistic model are presented in Table 3: life stress events and “parental relation styles” are both significantly associated with positive lymph-node status, adjusting for age and tumor burden. An optimal relation with at least one parent decreases the risk of positive lymph node (OR = 0.38; 95% CI: 0.17–0.85, *p* = 0.020), whereas sentimental life stress events increases the risk of positive lymph-nodes (OR = 3.18; 95% CI: 0.88–14.49), but the association was only borderline significant (*p* = 0.080).

**Table 3:** Results from multiple logistic model for the association with positive lymph node status. *InSitu* were excluded from the analysis. Significant P-values are indicated in bold, nearly significant P-values are indicated in italics.

		OR	Low 95% CI	Up 95% CI	P-values
Parental relation	Optimal vs other	0.38	0.17	0.85	**0.02**
Sentimental Life events	More than one vs other	3.18	0.88	11.49	*0*.*08*
Tumor burden		1.72	1.43	2.58	**0.01**
Age		1.02	0.97	1.08	0.34

## Discussion

In the present work, we asked 123 women who underwent surgery for primary breast cancer to complete two questionnaires measuring parental bonding and stress events throughout the life-span. The link between these variables and relevant prognostic factors in breast cancer was assessed. We found that in the absence of at least one optimal parental relationships the probability of having lymph node involvement significantly increased, controlling for main confounding factors. Furthermore, repeated stress in the relational area was borderline significantly associated with a greater risk of having lymph nodes involved. Axillary lymph node status is currently one of the most significant prognostic factors for patients with breast cancer [25].

Our data adds to previous evidence on the role of social support and stressful life events in breast cancer (see e.g., [3,7]) by suggesting that significant relations may have a pivotal role in determining resilience to disease. Our results are in line with evolutionary theories highlighting that adaption to the environment resides in the construction of social and affective relations, and thus that these aspects concurrently modulate also biological factors [26]. We hypothesize that early (as represented by non optimal parental bonding) and adult repeated relational stress may influence breast cancer prognosis and in particular lymph node involvement through HPA dysregulations, inflammatory responses and suppressed immune surveillance.

A number of studies demonstrated that stress during childhood can lead later in life to greater emotional (e.g., [27]) and physiological responses to fearful or challenging events [28], leading to an imbalance in the response of the HPA axis and to an increased activity of the autonomic nervous system. Higher attachment anxiety in adulthood is positively associated with a higher cortisol response to acute stress unrelated to attachment, and correlates negatively with the cortisol response to awakening [29]. Interestingly, abnormal diurnal cortisol patterns predict earlier mortality in breast cancer, independent from other known risk factors [30].

Murine models demonstrated that adequate maternal care in early critical periods increases, amongst others, the expression of the glucocorticoid receptor (GR) in brain regions involved in HPA activation [11]. This effect seems to be mediated by demethylation processes reversing initial hypermethylation of the promoter, that occur when maternal behaviors (such as tactile stimulation in rodents) are carried out. On the contrary, when maternal care is inadequate or epigenetic processes are inhibited in early critical periods, an hypermethylated state is maintained on genetic sites relevant for stress pathways [13]. Such a mechanism can explain how maternal care influences vulnerability to stress in the life span [11].

Stressful experiences induce the release of glucocorticoid and cathecolamines. These molecules seem to have a role in tumor angiogenesis by inducing the production of cytokines with a pro-angiogenic effect [3] e.g., vascular endothelial growth factor (VEGF). Interestingly, pro-angiogenic cytokines such as VEGF, have been related to lymph node involvement [3].

Non-optimal attachment can also have a role in determining pro-inflammatory responses. For instance, adverse childhood experiences are associated with higher levels of circulating IL-6, which is known to be a tumor-promoting factor involved in breast cancer development and progression [31]. In contrast, maternal warmth decreases production of IL-6 and activation of nuclear factor-kappa B (NFκB) in individuals exposed to stressful life conditions [32]. Similarly, relational stress was associated with greater IL-6 and NFκB activation in adolescents [33]. High serum IL-6 levels independently associate with lymph node involvement and lymphovascular invasion [34]. NFκB is a protein regulating transcription of DNA, linked to the stress response in humans together with cathecolamines and cortisol and is involved in the production of IL-6 [35]. Importantly, NFκB is associated with the migration of breast cancer cells producing metastases [36] and may thus partly explain the link between parental relations, sentimental stressful situations and lymph node involvement. Furthermore, NFκB regulates the expression of other inflammation-related factors such as cyclo-oxygenase-2, an enzyme catalyzing the synthesis of prostaglandins. COX-2 can increase angiogenesis in breast cancer and therefore can reduce the adherence of tumor cells to the extracellular matrix which results, as a first step, in lymph node involvement. Consistently, greater expression of COX-2 was also significantly associated with lymph node metastases [37].

An alternative, but not mutually exclusive, hypothesis to explain our results relies on psychological mechanisms. Insecure attachment styles seem to modulate referral to healthcare professionals. For instance, avoidant attachment is related to a reduced number of visits to healthcare professionals (controlling for number of symptoms; [38]). Therefore, insecure attachment may have led to a delayed diagnosis, thus influencing lymph node involvement. However, this hypothesis is less likely to explain our data. Since a delayed diagnosis should be represented also by a greater tumor mass, we would expect insecure attachment to be linked to tumor burden, which in turn would influence lymph node involvement. However this was not the case, since our analysis shows that: a) tumor burden is not related to parental attachment; and b) the association between parental attachment and lymph node involvement remains significant even when controlling for tumor burden.

Insecure parental attachment and sentimental stress can affect cancer prognosis also by promoting depression and detrimental health behaviors (which may be known risk factors for cancer, such as smoking or drinking). However, as our study specifically focuses on prognostic variables, an indirect association would be expected to impact more broadly on them. The fact that both parental bonding and sentimental stress point to the same prognostic factor suggests that a common mechanism may subtend this result.

The present study used retrospective measures to evaluate the quality of parental relations and the occurrence of stressful life events throughout the life span. Importantly, previous studies successfully used retrospective measures of stressful life events to evaluate association with breast cancer incidence (e.g., [39]). Despite the possibility of events’ recall being less reliable in the life-span, measures asking to recall past life events show consistent correlations with the outcomes of such events [40]. Therefore exploration of the whole life-span was preferred since accumulation of emotional stress may influence the development and prognosis of breast cancer [39].

## Conclusions

Our study suggests that non-optimal parental bonding and, possibly, stressful events in the sentimental area across the life-span are positively associated with lymph node involvement in breast cancer. Although dysfunctional parental relations and sentimental stress may not cause cancer *per se*, these factors could influence the underlying cellular and molecular processes that facilitate malignant cell growth and thus predispose to higher chances of metastatic disease.

Limitations concern the small sample size and the intrinsically observational nature of the study, which do not allow to draw definitive conclusions and define as causal the associations found.

Future studies should evaluate such associations with a larger sample size whether these variables may interact between each other or with known risk factors, and if they play a role also in cancer survival and mortality. Further research is needed to determine relevant physiological pathways involved in these associations.

## Ethical Standards

The authors assert that all procedures contributing to this work comply with the ethical standards of the relevant national and institutional committees on human experimentation and with the Helsinki Declaration of 1975, as revised in 2008.

## Supporting Information

S1 Dataset Variables for data analysis. (XLS)

S1 Table Frequencies and percentages of patients (excluding inSitu) by type of relation with the mother and the father. (DOCX)
