# Supplementary material for: Stress Exposure in Significant Relationships Is Associated with Lymph Node Status in Breast Cancer
Source: PLoS One. 2016 Feb 24;11(2):e0149443. doi: 10.1371/journal.pone.0149443 (PMC4766188; doi:10.1371/journal.pone.0149443)
Supplement: S1 Table — (DOCX) [file pone.0149443.s002.docx]

**S1 Table. Frequencies and percentages of patients (excluding inSitu) by type of relation with the mother and the father**.

|  | **Mother** | | **Father** | |
| --- | --- | --- | --- | --- |
|  | **Frequency** | **%** | **Frequency** | **%** |
| *Optimal Parenting* | 32 | 28 | 38 | 33 |
| *Affectionate Constraint* | 23 | 20 | 23 | 20 |
| *Affectionless Control* | 42 | 37 | 33 | 29 |
| *Neglectful Parenting* | 17 | 15 | 11 | 10 |
| *NA* | 1 | 1 | 10 | 9 |

*NA= not available*
